# Autophagy Dysfunction, Cellular Senescence, and Abnormal Immune-Inflammatory Responses in AMD: From Mechanisms to Therapeutic Potential

**DOI:** 10.1155/2019/3632169

**Published:** 2019-05-22

**Authors:** Shoubi Wang, Xiaoran Wang, Yaqi Cheng, Weijie Ouyang, Xuan Sang, Jiahui Liu, Yaru Su, Ying Liu, Chaoyang Li, Liu Yang, Lin Jin, Zhichong Wang

**Affiliations:** ^1^State Key Laboratory of Ophthalmology, Zhongshan Ophthalmic Center, Sun Yat-sen University, Guangzhou 510060, China; ^2^Eye Institute of Xiamen University, Fujian Provincial Key Laboratory of Ophthalmology and Visual Science, School of Medicine, Xiamen University, Xiamen 361102, China; ^3^Department of Ophthalmology, Dongguan People's Hospital, Dongguan 523059, China

## Abstract

Age-related macular degeneration (AMD) is a blinding disease caused by multiple factors and is the primary cause of vision loss in the elderly. The morbidity of AMD increases every year. Currently, there is no effective treatment option for AMD. Intravitreal injection of antivascular endothelial growth factor (anti-VEGF) is currently the most widely used therapy, but it only aims at neovascularization, which is an intermediate pathological phenomenon of wet AMD, not at the etiological treatment. Anti-VEGF therapy can only temporarily delay the degeneration process of wet AMD, and AMD is easy to relapse after drug withdrawal. Therefore, it is urgent to deepen our understanding of the pathophysiological processes underlying AMD and to identify integrated or new strategies for AMD prevention and treatment. Recent studies have found that autophagy dysfunction in retinal pigment epithelial (RPE) cells, cellular senescence, and abnormal immune-inflammatory responses play key roles in the pathogenesis of AMD. For many age-related diseases, the main focus is currently the clearing of senescent cells (SNCs) as an antiaging treatment, thereby delaying diseases. However, in AMD, there is no relevant antiaging application. This review will discuss the pathogenesis of AMD and how interactions among RPE autophagy dysfunction, cellular senescence, and abnormal immune-inflammatory responses are involved in AMD, and it will summarize the three antiaging strategies that have been developed, with the aim of providing important information for the integrated prevention and treatment of AMD and laying the ground work for the application of antiaging strategies in AMD treatment.

## 1. Introduction

**Figure 1:**
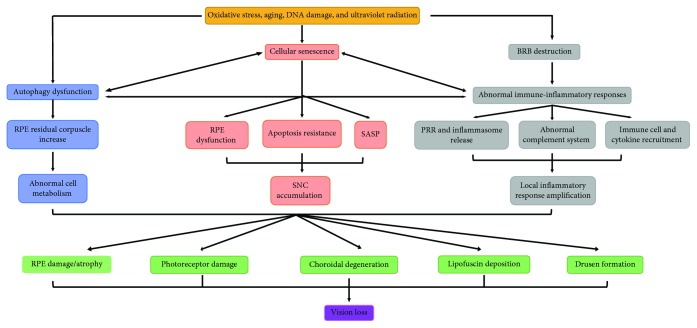
The relationship of RPE cell autophagy dysfunction, cellular senescence, and abnormal immune-inflammatory response in AMD. Oxidative stress, aging, DNA damage, and ultraviolet radiation can lead to RPE cell autophagy dysfunction, cellular senescence, and BRB destruction. Autophagy dysfunction results in the decreased clearance of RPE cells and increased intracellular residual corpuscles, which interferes with cell metabolism. Senescent RPE cells lead to cell dysfunction and promote the senescence of surrounding cells by secreting SASP. Moreover, SNCs are apoptosis resistant, failing to enter programmed cell death and aggregating instead. The destruction of the BRB could activate an abnormal immune-inflammatory response of the retina and lead to the release of PRRs and inflammasomes, the activation of immune cells and cytokines, and the activation of abnormalities of the complement system, which could further amplify the local inflammatory response. These factors interact with each other, causing lipofuscin deposition, drusen formation, RPE cell injury or atrophy, photoreceptor damage, choroid degeneration, and ultimately, loss of vision.

AMD is the leading cause of visual impairment among the elderly in western countries. Although AMD usually does not lead to complete blindness, it can result in the severe loss of central vision. A study estimated that, by 2020, 196 million people will be afflicted with AMD worldwide, increasing to 288 million people by 2040. As a result, the cost of AMD is predicted to increase to $59 billion over the next 20 years [1], suggesting that AMD is becoming a major public health issue. Currently, there is no effective treatment for 80% to 85% of the 30 to 50 million AMD patients worldwide [2]. AMD is a multifactorial blinding disease, and the exact cause of AMD is not yet clear. It has been previously demonstrated that oxidative stress [3], aging [4], DNA damage [5], and ultraviolet radiation [6] can lead to AMD by influencing the autophagy function of RPE cells, cellular senescence, and the immune-inflammatory response, which are closely related to each other in their mutual causation and promotion (Figure 1). Autophagy dysfunction results in the decreased clearance of cellular waste in RPE cells and increased intracellular residual corpuscles, which interfere with cell metabolism. Senescent RPE cells lead to cell dysfunction and promote the senescence of surrounding cells by secreting the senescence-associated secretory phenotype (SASP). Moreover, SNCs are apoptosis resistant, failing to enter programmed cell death and aggregating instead, further promoting the development of AMD. The blood-retinal barrier (BRB) has an immune privilege function. The destruction of the BRB could activate the immune-inflammatory response of the retina and lead to the release of pattern recognition receptors (PRRs) and inflammasomes, the activation of immune cells and cytokines, and abnormalities of the complement system, which could further amplify the local inflammatory response. The abovementioned factors interact with each other, causing lipofuscin deposition, drusen formation, RPE injury, or atrophy, which can lead to photoreceptor cell damage, choroid degeneration, and ultimately, loss of vision. These findings suggest that autophagy dysfunction in RPE cells, cellular senescence, and abnormal immune-inflammatory responses are involved in AMD pathogenesis and promote its progress. Here, we review the pathophysiological processes and interactions that are involved in AMD, with the aim of providing important information for the molecular, biological, and clinical research of AMD in the future.

## 2. Autophagy Dysfunction Leads to “Clearance System” Abnormalities

There are two major proteolytic systems that are responsible for maintaining cellular function: the proteasomal and lysosomal systems. Both systems remove irreversibly damaged proteins and recycle amino acids for protein synthesis [2, 7]. The autophagy-lysosome system is the most important of these two systems in RPE cells [2]. Autophagy can be divided into macroautophagy, microautophagy, and chaperone-mediated autophagy [8]. Macroautophagy, which is considered to be the major autophagic pathway and has been the most extensively studied type of autophagy, is mediated by the formation of an autophagosome, a double-membrane vacuole that contains the materials targeted for degradation (cargo). The autophagosome carries cargo to and combines with the lysosome to form the autolysosome, in which the final degradation of cargo occurs. This process requires the participation of a series of autophagy-related proteins (Atgs). Although observation of the double-membraned structure by transmission electron microscopy (TEM) is the gold standard for autophagy detection, it is necessary to assess the expression levels of LC3 II/LC3 I, p62/SQSTM1, and Atgs to estimate the level of autophagy activity [9]. The photoreceptor outer segments (POS) are composed of dense discs. Proteins are synthesized in the inner segments and transported to the outer segments through ciliary ligation structures to form new discs. Therefore, the POS are being continuously renewed. Once the discs have been internalized, autophagosomes move from the apical to the basal surface, where the cargo is degraded. This process can be divided into four distinct stages: recognition and attachment of the POS discs, POS disc ingestion, the formation of the autophagosome and its fusion with the lysosome, and degradation [10–13]. RPE cells are the most active autophagic cells in the whole body. Near the retinal fovea in primates, each RPE cell serves approximately 40 rod cells, and up to 10% of the POS are digested on a daily basis [13–15]. If autophagic dysfunction occurs in RPE cells, the accumulated POS cannot be degraded, which is accompanied by lipofuscin deposition and drusen formation and, subsequently, leads to the deaths of photoreceptor cells, vision loss, and the accelerated development of AMD [8, 16].

Studies have shown that, compared with those of the normal population, the RPE cells of AMD patients demonstrate increased numbers of autophagosomes, decreased LC3 II/I concentrations, decreased autophagy flow, and increased vulnerability to oxidative stress, indicating that autophagy dysfunction in RPE cells is involved in AMD [17]. The RB1CC1/FIP200 gene is involved in the induction of autophagy. The deletion of RB1CC1/FIP200 resulted in multiple autophagy defects, including a decreased ratio of LC3 II/LC3 I concentrations, the accumulation of autophagy-targeted precursors, and increased numbers of mitochondria. Age-related degeneration of RPE cells was also observed, accompanied by the formation of atrophic patches, the subretinal migration of activated microglial cells, the sub-RPE deposition of inflammatory and oxidatively damaged proteins and drusen, and occasional foci of choroidal neovascularization [18]. The RPE-specific deletion of Atg5 or Atg7 in mice induced autophagy deficiency. Markers of oxidatively damaged proteins and DNA were found to accumulate in RPE cells. Retinal degeneration was also observed in 35% of the Atg5^ΔRPE^ mice and 45% of the Atg7^ΔRPE^ mice aged 8 to 24 months old. In addition, the degeneration severity increased with age while the POS thickness decreased. Early AMD-like RPE defects were found in all the Atg5^ΔRPE^ and Atg7^ΔRPE^ mice starting at 13 months, including uneven RPE thickness, RPE hypertrophy/hypotrophy, pigmentary irregularities, choroidal neovascularization, and necrosis [19]. The visual cycle is fundamental to vision. RPE utilizes all-*trans* retinol (ROL) to synthesize the chromophore 11-*cis* retinal (RAL), which is then shuttled across the interphotoreceptor matrix to POS by the interphotoreceptor retinoid-binding protein (IRBP). Within the POS, 11-*cis* RAL is bound to G protein-coupled receptors (opsins) to form a light-sensitive visual pigment. Under light stimulation, 11-*cis* RAL transforms into an all-*trans* configuration, altering the three-dimensional structure of the opsin protein and activating the phototransduction signaling cascade. All-*trans* RAL then releases from the opsin protein, transforms into all-*trans* ROL, and is transported back to the RPE to be recycled back into 11-*cis* RAL. The Atg5^ΔRPE^ mice showed abnormal POS degradation and decreased visual cycle activity [20] while the 11-*cis*-RAL content was normal in Atg7^ΔRPE^ mice, and only abnormal RPE homeostasis was observed [16]. During this process, Atg5-dependent autophagy required the participation of Beclin1 [20].

Lipofuscin is a kind of photosensitizer and spontaneously oxidative substance, which can increase mitochondrial stress and irreversibly inhibit lysosomal protease activity following light irradiation, leading to RPE cell damage. Once formed, lipofuscin cannot be degraded by proteasomal or lysosomal enzymes or be transferred out of cells by extracellular secretion [13]. The accumulation of lipofuscin in RPE cells is one of the factors that leads to AMD [2]. A2E is the primary spontaneous fluorophore of lipofuscin. In retinal diseases, A2E oxidation products are involved in complement activation and inflammation [16, 21]. The combined use of A2E with the autophagy inhibitor 3-methyladenine (3-MA) resulted in the death of the RPE cells and increased reactive oxygen species (ROS) production [22]. Research has shown that the inhibition of autophagy increases lipofuscin-like autofluorescence (LLAF) while the activation of autophagy reduces it [14], suggesting that improving the autophagy levels in RPE cells can reduce lipofuscin accumulation, thus delaying the development of AMD.

Oxidative stress, one of the pathogenic factors of AMD, can mediate reactions to DNA damage, alter autophagy levels, and regulate cellular senescence [3]. Oxidative stress can induce electron leakage from the mitochondrial electron transport chain, followed by the formation of hydroxyl radicals and peroxides. The central retina is vulnerable to exposure to an exceptionally high burden of oxidative stress, which increases during aging. Sustained oxidative stress leads to impaired autophagy, protein accumulation, inflammatory response activation, and the formation of the AMD pathological phenotype [13]. The upregulation of autophagy by rapamycin decreased the oxidative stress-induced generation of ROS, whereas the inhibition of autophagy by 3-MA or by the knockdown of either ATG7 or BECN1 increased ROS generation, exacerbated the oxidative stress-induced reduction of mitochondrial activity, reduced cell viability, and increased lipofuscin concentrations [7]. Glucosamine (GlcN) is a naturally occurring amino monosaccharide with immunosuppressive effects that can inhibit the inflammatory response and the epithelial-mesenchymal transformation of RPE cells and protect retinal glial cells from oxidative stress. GlcN can decrease the native POS-derived LLAF through the induction of autophagy, partly through the AMPK-mTOR pathway [23]. Melatonin is an antioxidant that scavenges free radicals and has anti-inflammatory, antitumor, and antiangiogenic effects. Melatonin upregulates the expression of LC3 II and Beclin1 and downregulates p62 to promote autophagy [24]. The abovementioned evidence suggests that autophagy plays a key role in protecting RPE cells from oxidative stress and lipofuscin deposition.

## 3. RPE Cellular Senescence Leads to Cell Dysfunction and Promotes the Senescence of Neighboring Cells

**Figure 2:**
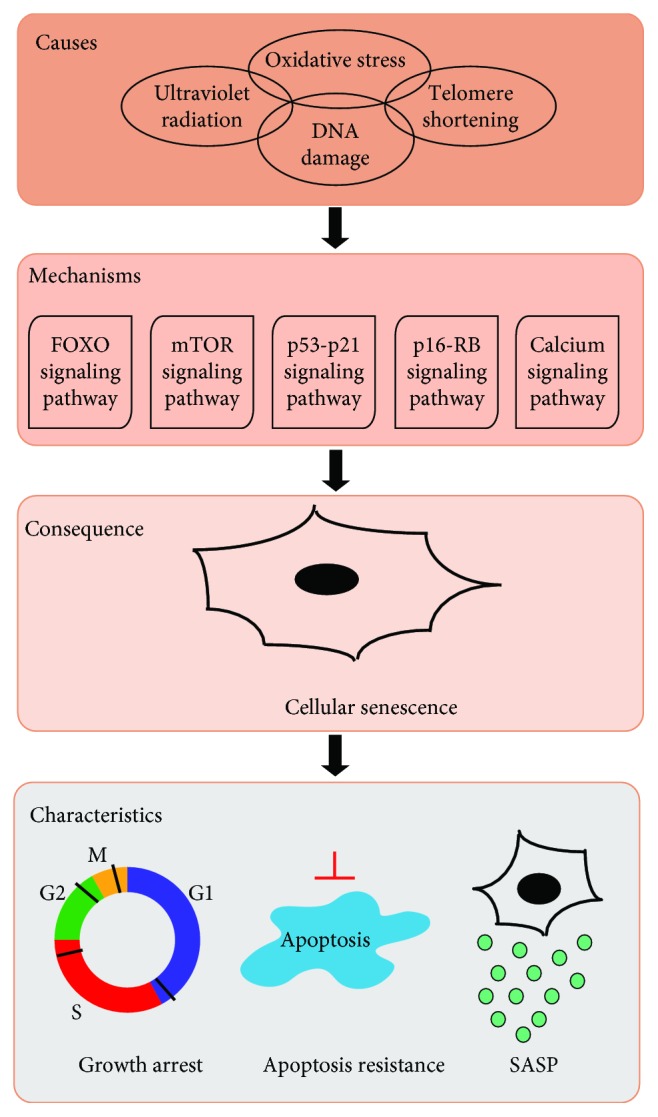
An overview of cellular senescence. A variety of stimuli, such as oxidative stress, DNA damage, ultraviolet radiation, and telomere shortening can induce a series of reactions, including the activation of the FOXO signaling pathway, the mTOR signaling pathway, the p53-p21 signaling pathway, the p16-Rb signaling pathway, and the calcium signaling pathway, ultimately leading to cellular senescence. SNCs have several primary characteristics, such as growth arrest, apoptosis resistance, and SASP secretion.

Cellular senescence was first mentioned by Hayflick and Moorhead in 1961 [25]. Aging is characterized by the declining ability to maintain homeostasis in multiple tissues and limited somatic cell division. These inabilities can be observed at the cellular level as the dysfunction of self-repair and renewal, cell cycle arrest, and the appearance of SNCs [26]. Changes in the immune system function and the apoptotic resistance of SNCs result in SNC accumulation [27], causing a range of age-related diseases, such as Alzheimer's disease, osteoarthritis, pulmonary fibrosis, and AMD [28, 29]. The p16^INK4A^-pRB and p53-p21^CIP1/WAF1^ pathways are primarily involved in the mechanism of cellular senescence (Figure 2) [26, 30]. The activation of p53 upregulates p21^CIP1/WAF1^ and inhibits the cell cycle proteins cyclin A, E, and D. The activation of the pRB pathway is mediated by p16^INK4A^, which is independent of p53. p16^INK4A^ inhibits Cyclin A-, Cyclin E-, and Cyclin D-dependent kinase complexes, which normally phosphorylate and inactivate pRB. Dephosphorylated pRB represses the G_1_/S transition by sequestering E2F transcription factors, thereby inhibiting E2F-dependent gene expression [30]. Although SNCs are blocked at the G_0_/G_1_ or G_2_/M stages and cannot undergo cell division, they can still exist in a long-term metabolically active state, accompanied by the upregulation of inflammatory factors, chemokines, matrix remodeling proteases, and growth factors, which are collectively referred to as SASP. SASP in the tissue microenvironment promotes a series of inflammation cascades and accelerates the senescence of surrounding cells [28, 31], which is related to age-related inflammatory reactions, metabolic disorders, stem cell dysfunction, and chronic diseases [29]. The SASP components vary depending on cell type and senescence trigger factors. The proinflammatory cytokines IL-1*α*, IL-1*β*, IL-6, and IL-8 are classical SASP components. Multiple genes are involved in the biological regulation of SASP, including NK-*κ*B, p38-MAPK, mTOR, and GATA4 [28].

Cellular senescence can be divided into two types: replicative senescence (RS) and stress-induced premature senescence (SIPS) [32, 33]. Recently, scholars have proposed a third type, developmentally programmed senescence (DPS) [31]. RS is caused by telomere shortening during cell replication [28]. A telomere is a type of complex composed of proteins and nucleotides containing TTAGGG repeats found at the ends of eukaryotic chromosomes [33]. To protect against genomic instability caused by shortened telomeres, DNA damage response (DDR) activates to induce a series of cascade reactions, including ATM/ATR-mediated p53-p21^CIP1/WAF1^ and p16^INK4A^-pRB pathway activation, cell cycle arrest, and apoptosis. Precipitating factors for SIPS include oxidative stress, oncogenes, genotoxic damage, chemotherapy, and viral infection [26, 30, 31]. DPS can occur anywhere during the process of mammalian embryo formation. Interestingly, DNA damage markers and the DNA damage-dependent kinase ATM/ATR were not detected in DPS cells. Megakaryocytes and NK cells are the only adult cell types that appear to undergo DPS [31]. Currently, the following markers are used to determine cell senescence: (1) altered cellular morphology (often enlarged, flat, multivacuoled, and multinucleated); (2) increased Senescence *β*-Galactosidase (SA-*β*-GAL) activity; (3) the accumulation of DNA damage foci; (4) the accumulation of senescence-associated heterochromatic foci (SAHF) and other chromatin modifications; (5) chromosomal instability; (6) the induction of SASP; and (7) the altered expression of senescence-related genes (i.e., p53, p21^CIP1/WAF1^, p16^INK4A^, pRB, and cyclin-dependent kinases) [31, 32, 34].

Cellular senescence is one of the pathogenic factors underlying AMD. The senescence-accelerated OXYS rat is an animal model of AMD that can spontaneously undergo an AMD-like retinopathy, including RPE degeneration, loss of photoreceptors, and the decreased expression of vascular endothelial growth factor (VEGF) and pigment epithelial-derived factor (PEGF) [35, 36]. Chorionic capillary membrane attack complex (MAC) deposition can cause chorionic capillary degeneration and RPE atrophy, leading to dry AMD. Senescent chorioretinal endothelial cells are significantly stiffer than normal cells, which correlates with higher cytoskeletal Rho activity and more susceptibility to MAC injury [37]. Each microglial cell possesses ramified, branching processes that exhibit rapid, constitutive motility, which enables the cell to effectively survey the extracellular milieu in its vicinity. While microglial somata are evenly spaced and relatively stationary in the uninjured state, following focal injury, microglia promptly polarize their processes and migrate in the direction of the injury to cluster around the injury site. However, a thickened glial layer, decreased branch numbers, shortened lengths, and slowed movement may occur during aging, which can lead to changes in homeostasis and promote long-term retinal neuroinflammation, as reflected by increased levels of complement C3 and CFB [38], further promoting AMD progress.

Telomere shortening of RPE cells is also one of the characteristics of AMD. Late passage RPE cells from primary culture demonstrated a reduced capacity for cellular division, which could be caused by telomere shortening [39]. A2E could contribute to RPE cellular senescence, accompanied by telomere deprotection and deletion, and telomerase overexpression rescued A2E-mediated RPE cellular senescence, indicating that telomere dysfunction plays an important role in A2E-based RPE cellular senescence [40].

Long-term and chronic oxidative stress, which are pathogenic factors of AMD, can be generated by cigarettes, hydrogen peroxide (H_2_O_2_), *tert*-butyl hydroperoxide (TBHP), and light and can result in the premature aging of RPE cells, which is characterized by increases in ROS and SA-*β*-GAL activities, higher expression levels of p53, p21^WAF1/CIP1^, p16^INK4A^, and SASP factors, the accumulation of p-*γ*H2AX foci and 8-OHdG DNA damage lesions, mitochondrial dysfunction, increased VEGF, and decreased CFH [41–44]. HTRA1 is closely related to AMD and can accelerate H_2_O_2_-mediated RPE senescence through the p38 pathway [44]. Many studies have shown that inhibiting oxidative stress can reduce RPE senescence. For instance, fullerenol, an effective free radical scavenger and antioxidant, can strengthen the antioxidant reaction of RPE and alleviate DNA damage by activating SIRT1 and downregulating p53 and p21^CIP1/WAF1^ levels [42]. SIRT1, a member of the SIRT family, is the primary longevity gene that prolongs life and reduces cancer-associated metabolic syndrome [45]. Humanin has been shown to have anti-inflammatory and cell-protective effects in a variety of cell types. Humanin alleviates RPE oxidative stress damage and senescence by phosphorylating STAT3 and inhibiting caspase-3 activation [43]. Both SIRT1 and STAT3 have protective effects on RPE cells. Under oxidative stress, SIRT1 is downregulated, while STAT3 is upregulated, and the regulation of STAT3 is independent of SIRT1 [46]. PCG1*α*, a transcription regulator, is involved in mitochondrial metabolism and is associated with many age-related diseases. PCG1*α* protects RPE cells from oxidative stress by upregulating antioxidant enzymes and DDR and is regulated by AMPK and SIRT1 during the process of posttranscriptional modification and activation [47].

Interfering with the proageing effects of SNCs, either by eliminating SNCs entirely or by shutting down their secretory machinery, is now being considered as a potential strategy for treating diseases associated with aging. The selective removal of SNCs can prolong life and reduce some side effects of drugs, such as bone marrow suppression, cardiac dysfunction, and toxic effects. Broadly, three strategies have been used for the selective elimination of SNCs (Table 1): (1) immune-mediated SNC clearance, which utilizes antibodies targeting senescence-specific surface antigens to clear SNCs; (2) senescent cell lysis (senolysis), which leads to the death of SNCs by activating apoptotic pathways; and (3) SASP neutralization, including the inhibition of SASP-related signaling cascades, interference with the SASP secretome, and the inhibition of individual secretion factors. Among these, senolysis holds the most therapeutic promise. Currently, no relevant strategies for SNC clearance has been applied to AMD treatment [27, 28].

### 3.1. Immune Surveillance Mediates SNC Clearance

NK cells are a component of the innate immune system. One of the receptors responsible for NK cell activation, the NKG2D receptor, has been implicated in the interaction between NK cells and SNCs during tumorigenesis, tumor therapy, and tissue injury. The NKG2D receptor recognizes the ligands MICA/B and ULBP1-6 on the surface of SNCs to recruit NK cells for immune surveillance regulation. For example, NK cells mediate the clearance of SNCs during liver fibrosis [48]. Intercellular adhesion molecule 1 (ICAM-1) is commonly present on the surface of SNCs and might cooperate with NKG2D ligands to amplify the cytotoxicity of NK cells [49]. P53-positive SNC accumulation mediates the generation of CCL2, 3, 4, and 5 and CXCL1 and 2. These cytokines activate NK cells and recruit immune cells to clear senescent tumor cells [50]. Macrophages are tissue-resident phagocytes equipped with a complete arsenal of pathogen recognition receptors that enable them to sense potential risks. Upon stimulation, macrophages acquire context-dependent phenotypes by undergoing either classical M1 or M2 polarized activation. For example, senescent hepatic stellate cells (HSCs) release SASP factors such as IFN-c and IL-6 that skew macrophages toward the M1 state to attack HSCs during liver diseases [49]. Furthermore, the macrophage scavenger receptor CD36 (oxPC_CD36_) is enriched in both atherosclerotic plaques and on SNC membranes, serving as a critical participant in macrophage recognition. This receptor produces a surface accessible phagocytic “eat me signal” to facilitate the recognition of SNCs and oxidized lipoproteins as part of its immune surveillance function [51]. In addition to NK cells and macrophages, monocytes also participate in immune-mediated SNC clearance. Senescent fibroblasts can stimulate monocyte production in the bone marrow via the robust secretion of two inflammatory SASP components, GM-CSF and G-CSF. These SASP factors can direct monocytes to the SNCs. Then, SNCs promote the differentiation of these monocytes into macrophages via the secretion of M-CSF [49]. Ipilimumab, an antibody that enhances cytotoxic T cell activation through the blockade of the cytotoxic T-lymphocyte-associated protein 4 (CTLA-4) receptor or the antiprogrammed cell death protein 1 (PD1), can activate the immune surveillance response that has been suppressed in cancer cells [52]. In addition, antibodies against senescence-specific surface antigens, such as CD44 in endothelial cells, could induce a direct immune response or deliver cytotoxic drugs to senescent lesions to mediate SNC clearance [28].

### 3.2. Senolysis Mediates SNC Apoptosis

Dasatinib (D), a tyrosine kinase inhibitor, can inhibit cell replication and migration and induce apoptosis [53]. Quercetin (Q) is a flavonoid complement. D alone can downregulate p21^CIP1/WAF1^, clearing senescent fat precursor cells. Q clears senescent human endothelial cells and mouse bone marrow mesenchymal stem cells (BM-MSCs). D+Q can downregulate p21^CIP1/WAF1^, BCL-Xl, and PAI-2 and effectively clear senescent fibroblasts (MEFs) [54]. Bleomycin induces the age-dependent accumulation of senescent MEFs in the lungs, further leading to pulmonary fibrosis. D+Q treatment can clear SNCs mediated by bleomycin and downregulate p16^INK4^ and the SASP components MCP-1, IL-6, MMP-2, and TGF-*β* [55]. Transplanting relatively small numbers of SNCs into young mice caused persistent physical dysfunction and spread cellular senescence to host tissues. The application of D+Q decreased the numbers of naturally occurring senescent cells and decreased the secretion of the SASP components IL-6, IL-8, MCP-1, PAI-1, and GM-CSF. Moreover, the administration of D+Q to both senescent cell-transplanted young mice and naturally aged mice alleviated physical dysfunction, increased posttreatment survival, and reduced mortality hazard [56]. The application of SNC removal has not been studied in AMD, but studies have shown that Q can protect RPE cells from oxidative stress through its antioxidant effects [57], inhibit choroid neovascularization [58], and upregulate BCL-2 while downregulating Bax [59, 60]. ABT-263, an activator of the mitochondrial apoptosis pathway, can inhibit Bcl-2, BCL-W, and BCL-Xl. ABT-737 can inhibit BCL-W and BCL-Xl. Both ABT-263 and ABT-737 are involved in removing senescent MEFs from pulmonary and human umbilical vein endothelial cells (HUVECs) [27, 28, 53]. FOXO4 is elevated in SNCs and maintains their viability. FOXO4 exists in the PML body and combines with p53 DNA-SCARS. DRI is a kind of polypeptide that has been used in phase I clinical trials against solid tumors. Researchers have designed and synthesized FOXO4-DRI to effectively and powerfully target SNCs and mediate p53-dependent apoptosis to remove SNCs by destroying PML/DNA-SCARS in SNCs and competing with FOXO4 to bind to P53. At the tissue level, FOXO4-DRI alleviated hepatic dysfunction induced by chemotherapy and improved the frailty properties and renal functions of both xpdTTD/TTD mice (an animal model of premature aging) and naturally aged mice [61]. In another study, SNCs were marked using p16^INK4A^. An aging BubR1H/H mouse model containing INK-ATTAC lines was established, which showed shortened lifetime, lordosis, cataracts, and the aggregation of p16^INK4A^-positive cells. AP20187, a synthetic drug that induces apoptosis through cell membrane dimerization, was given to the BubR1H/H mice. AP20187 activated INK-ATTAC, which aided the accurate identification of p16^INK4A^-positive SNCs and effectively cleared them while not affecting normal cells, reducing the senescent phenotypes of adipose tissue, skeletal muscle, and the eye [62].

### 3.3. SASP Neutralization Mediates the Weakened Proaging Effect of SNCs

SASP inhibitors include rapamycin, metformin, and JAK1/2 inhibitors. Rapamycin reduces the secretome of inflammatory factors in SNCs by inhibiting mTOR1 [28, 53], playing a role in prolonging lifespan, and reducing age-related fatty tissue loss, heart failure, and cognitive impairment [29]. Metformin inactivates NF-*κ*B and reduces SASP component levels by inhibiting the phosphorylation of I*κ*B and IKK*α*/*β* [63]. JAK is a tyrosine kinase that is highly active in SNCs [64]. Using siRNA or JAK inhibitors to inhibit the secretion of the SASP factors IL-6, IL-8, and MCP-1 in both senescent adipose progenitor cells and HUVECs improved the physical functions of elderly mice and alleviated insulin resistance and stem cell dysfunction [29, 65]. UBX0101, a senolytic molecule, can combine with MMP-13, IL-6, and IL-1*β* [27]. The intra-articular injection of UBX0101 selectively eliminated SNCs after anterior cruciate ligament transection (ACLT), attenuated the development of posttraumatic OA, reduced pain, and increased cartilage development [66].

Among the three aging-therapy strategies, senolysis holds the most therapeutic promise for two reasons. First, the permanent removal of SNCs leads to the durable abolishment of deleterious SASP components. Second, once SNCs are eliminated, there is no risk of tumorigenic “escape” from senescence, which may be possible if SNCs are permitted to linger indefinitely [27]. However, almost all drugs have off-target and bystander effects. For example, the removal of p16^INK4A^-positive cells by senolytic drugs has the following problems: (1) not all senescent cells necessarily have increased p16^INK4A^ expression; (2) not every cell with substantial p16^INK4A^ expression is senescent; (3) targeting aging mechanisms can phenocopy the effects of genetic or pharmacological SNC clearance without actually affecting SNCs; and (4) hypothetically, the genetic clearance of p16^INK4A^-positive cells could have the same effects on a particular downstream phenotype as a drug that affects that downstream phenotype directly, without affecting truly senescent p16^INK4A^-positive cells. To determine whether senolytic drugs actually cause the alleviation of senescence-associated phenotypes due to SNC clearance requires following a modified set of Koch's postulates, which are the following: (1) individual SNCs or transplanted SNCs must have a senescent phenotype; (2) the clearance of SNCs genetically or pharmacologically must be associated with the alleviation of the phenotype; and (3) the effects on the phenotype should persist even after the drug has been removed [29].

SNC clearance can alleviate the further senescence and tissue damage of surrounding cells, thereby delaying disease progression. However, SNC clearance methods are not universal and depend on the types of cells and diseases, which complicates the treatment prospects [67]. At present, the scavenging of SNCs has not been applied to AMD, and a large amount of in-depth research is needed to confirm whether SNC clearance is feasible for AMD prevention and treatment.

## 4. Abnormal Immune-Inflammatory Responses Are Pathogenic Factors for AMD

Inflammation is the body's response to cell and tissue damage and occurs through a series of processes that are designed for the eventual clearance of pathogens and the repair of damaged tissue. Acute inflammation is a short-term process that involves leukocyte infiltration, the removal of the trigger, and tissue repair. Chronic inflammation is a prolonged response that can result in tissue injury or destruction if the inciting trigger is not neutralized [1]. Inflammation is a common cause of age-related diseases. Chronic inflammation is involved in AMD [68], and the “immune-inflammation” model of AMD has been broadly accepted [69]. The retina is a purported “immune privileged” site, protected by the BRB, ocular anti-inflammatory and anti-immune proteins, and the anterior chamber-associated immune deviation [70]. Once these protective mechanisms are destroyed, abnormal immune and inflammatory responses occur, accelerating the development of AMD.

### 4.1. PRR and Inflammasome Release Mediates a Chronic Inflammatory Response

The body recognizes exogenous pathogens and endogenous risk factors through pattern recognition receptors (PRRs) that sense microbes through conserved molecular structures called pathogen-associated molecular patterns (PAMPs). PPRs include the Toll-like receptors (TLRs), the nucleotide-binding oligomerization- (NOD-) like receptors (NLRs), the RIG-I-like receptors (RLRs), the C-type lectin receptors, and advanced glycosylation end product (AGE) receptors (RAGE) [71]. When these receptors bind to their corresponding ligands, inflammasomes in cells activate, causing the release of inflammatory mediators. NLRP3 is a member of the NLR family, which can assemble into a large oligomeric structure through the recruitment of an adaptor protein, ASC, and procaspase-1 and can subsequently produce mature IL-1*β* and IL-18 through a two-step process. Classically, the first step, referred to as inflammasome priming, involves the NF-*κ*B-mediated synthesis of the inactive precursors pro-IL-1*β* and pro-IL-18 in response to the recognition of a specific ligand by its corresponding PRR and the upregulation of inflammasome components, including NLRP3. A second signal is required for NLRP3 oligomerization, the recruitment of ASC and procaspase-1, and the subsequent cleavage of procaspase-1 into its active form, leading to the processing of pro-IL-1*β* and pro-IL-18 and eventually to the release of the mature cytokines IL-1*β* and IL-18 [72]. High expression levels of NLRP3, IL-1*β*, and IL-18 can be detected in the photoreceptor and RPE cells of AMD patients [73]. Mitochondrial dysfunction, oxidative stress, and drusen can overly activate NLRP3 [13]. Laser-induced choroidal neovascularization (CNV), a mouse model of wet AMD, is exacerbated in NLRP3^−/−^ mice [74]. However, due to the existence of several nonspecific commercially available anti-NLRP3 antibodies that questions current interpretation of results reporting NLRP3 expression and upregulation in the RPE cells of AMD patients, the problems with NLRP3 activation in RPE cells and the measurements of this process have been signalized recently [75]. The study argues that RPE cells may not contain meaningful amounts of NLRP3 to contribute to diseased states and suggests that if NLRP3 is implicated in AMD, it is more likely to be related to immune cells, either resident or infiltrating. Thus, further evidence is required to characterize the presence and source and activation of pro-IL-18 in AMD.

Alu is the most abundant transposable element, which is transcribed into Alu RNAs, and the accumulation of Alu RNAs has been confirmed to be related to AMD [76]. Alu RNAs, by reducing DICER1, can activate the inflammasome in RPE cells and increase IL-18 levels, leading to geographic atrophy. Additionally, DICER1 deficiency combined with Alu RNA accumulation resulted in increased IL-18 levels, which led to RPE cell death via the activation of caspase-8 through a Fas ligand-dependent mechanism [1].

In addition to RAGE, some substances that are secreted by dead cells and damaged tissues are also receptors for AGEs, including amyloid *β*-protein (A*β*). In the central nervous system, the accumulation of A*β* is associated with the activation of neurodegenerative and inflammatory pathways. In the ocular system, A*β* upregulates IL-1*β*, IL-18, and TNF-*α* in RPE cells. The intravitreal injection of A*β* can activate inflammation [77]. AGEs accumulate with aging. AGE deposits were found in drusen, and studies have suggested that AGE plays a role in the promotion of oxidative stress, apoptosis, and lipofuscin accumulation. The in vitro incubation of RPE cells with AGEs resulted in the upregulation of the anti-inflammatory cytokines IL-10, IL-1ra, and IL-9 and the proinflammatory cytokines IL-4, IL-15, and IFN-*γ*, while other proinflammatory cytokines, such as IL-8, MCP-1, and IP10, were downregulated, suggesting a that parainflammation state occurred under AGE stimulation [78]. Parainflammation, a state between normal and inflammatory responses, is thought to be beneficial for the host. However, if tissue malfunction is sustained over long periods, parainflammation can become chronic and maladaptive. In AMD, the balance between stress-induced damage and parainflammation is often disrupted due to environmental and genetic factors, resulting in a chronic inflammatory state [79]. One explanation for the shift from early AMD to late AMD is that triggers can switch an aging homeostatic parainflammatory response into a persistent low-grade inflammatory response, leading to the loss of RPE cells and/or pathological angiogenesis [80]. All of these data suggest that PRRs and inflammasomes have close associations with AMD.

### 4.2. Abnormal Complement System Amplifies Cascade Reaction

The complement system is part of the host innate immune system that enables many essential functions, including the following: (1) the opsonization and lysis of microorganisms, (2) the recruitment of inflammatory cells, (3) the removal of dead cells, (4) the regulation of antibody production, and (5) the removal of immune complexes. There are three classic complement pathways: the classical pathway, the mannan-binding lectin (MBL) pathway, and the alternative pathway. All of these pathways ultimately lead to the formation of the cytolytic MAC [68]. The complement system is a double-edged sword for the retina. A low level of complement activation is beneficial to immune privilege, and RPE cells can produce complement components belonging to the classical pathway and the alternative pathway such as membrane-binding regulators and soluble regulators to prevent excessive complement pathway activation [81]. However, if the complement pathway is overactivated, it can damage retinal tissues and lead to the chemotactic aggregation of immunocytes. Studies have shown that plasma concentrations of the activation products C3a, C3d, Ba, Bb, C5a, and CFH are high in AMD patients. Similar alterations were observed for C3, C3d, and C5-9 in drusen. C-reactive protein (CRP) and C5 were primarily found in dry AMD, while C3a and C5a were primarily found in wet AMD [82, 83]. CRP is a biomarker of acute inflammation and plays an essential role in the innate immune response to tissue injury and/or infection, inducing complement activation via the alternative pathway [69]. Complement factor H (CFH) and HTRA1/ARMS polymorphisms contribute to more than 50% of the genetic risk for AMD [84]. CRP damages cells and tissues by binding to DNA or phosphocholine that has been exposed in injured cells and activating the classical complement pathway, resulting in the formation of the C3 convertase, which generates C3b. By binding the inhibitor CFH, C3b promotes the complement cascade and the formation of the C5 convertase. Polymorphisms in the complement components C2 and Factor B (CFB) are protective for AMD. C2 is a component of the classical complement pathway, and CFB is involved in the alternative pathway. Genetic and functional data suggest that this protective effect is more likely to be mediated by mutations in the CFB gene than by mutations in the C2 gene. The AMD-associated CFB variants modulate the activation of the alternative complement pathway and, therefore, may lead to an overall deregulation of the complement system, which may lead to the further amplification and inflammation of the complement cascade [83]. The complement system is also closely associated with inflammation. The inflammasome can be activated by a number of triggers, notably C5b-9 and C3a. The C3a-mediated ATP release prompts the P2X7 receptor to bind to and activate NLRP3 [1]. Thus, the complement system and the inflammasome can synergize to promote AMD progression after abnormal activation.

### 4.3. The Activation of Immune Cells and Cytokines Promotes Inflammation

Immune cells in a normal retina include microglial cells (MCs), macrophages, and dendritic cells. MCs play roles in neuronal homeostasis and immune surveillance, which are normally absent from the outer retina but can infiltrate into the subretinal space and become activated during aging and AMD, likely to support the RPE cells and clear age-related debris. However, MCs may also induce oxidative stress and promote further degeneration. Two chemokines, CX3CL1 and CCL2, and their respective receptors, CX3CR1 and CCR2, play important functions for the recruitment of macrophages/microglia to tissue lesions [70]. In CX3CR1-deficient (CX3CR1^−/−^) mice, MCs and drusen-like deposits accumulated subretinally with age [85]. In CX3CR1^−/−^/CCL2^−/−^ (double knockout) mice, AMD-like retinal lesions developed, characterized by abnormal RPE cells, drusen, photoreceptor atrophy, and choroidal neovascularization [86]. Macrophages, a predominant cell type associated with chronic inflammation, are the most prominent inflammatory cells observed in AMD tissue, outnumbering subretinal MCs and lymphocytes in AMD eyes. Macrophages secrete a wide range of cytokines, chemokines, complement factors, and growth factors, all of which depend on the inciting stimuli, macrophage subtype, and location. Macrophages can display as different subclasses, namely, the M1 and M2 macrophages. M1 macrophages have been shown to be proinflammatory, with an IL-12^high^, IL-23^high^, and IL-10^low^ phenotype, while the M2 macrophages are relatively anti-inflammatory with IL-12^low^, IL-23^low^, and IL-10^high^ phenotype. In addition, CXCL9, CXCL10, and CXCL11 represent M1 chemokines, and CCL17 and CCL22 represent M2 chemokines [70]. CXCL11 is strongly immunoreactive and associated with drusen. The upregulation of CXCL1, along with viperin and RSAD2, may play a role in driving the inflammatory response via the NF-*κ*B and JAK-STAT pathways [78]. IL-17 has previously been shown to be involved in inflammation and autoimmune diseases and can be produced by T cells and innate immune cells (ILC). The IL-17 cytokine family includes six members named IL-17A-F. IL-17A, produced primarily by Th17 cells, is the primary subfamily member. Under specific conditions, other inflammatory cells such as neutrophils and even macrophages can produce IL-17A. IL-17A homodimers bind IL-17 receptor C (IL-17RC)/IL-17RA heterodimers, which are involved in proinflammatory responses. IL-17 produced by *γδ*T, and ILC promoted experimental intraocular neovascularization during laser-induced CNV in mice. Additionally, there was a greater increase in the expression levels of IL-17RC in the blood of siblings with AMD compared to that in the blood of their respective siblings without AMD, and increased levels of IL-17RC can cause damage to photoreceptors [70, 87]. Therefore, immune cells can secrete inflammatory cytokines, further promoting retinal inflammatory responses.

## 5. Autophagy Dysfunction, Cellular Senescence, and Abnormal Immune-Inflammatory Responses Can Promote or Inhibit Each Other

Autophagy dysfunction, cellular senescence, and abnormal immune-inflammatory responses interact with each other. Autophagy dysfunction accompanied by lipofuscin accumulation and ROS increases, can activate inflammatory reactions, further promoting long-term and chronic cascade inflammation and accelerating RPE cell senescence [13]. Nrf2, a basic leucine zipper transcription factor, regulates a coordinated transcriptional program that allows cellular redox homeostasis while protecting the cell from oxidative injury [1]. Nrf2 physically interacts with a negative regulator Keap1, which targets the Nrf2 protein for ubiquitination and proteasomal degradation within the cytoplasm, thus limiting its activity. However, under oxidative stress, Keap1 undergoes a conformational modification and releases Nrf2, allowing it to undergo translocation to the nucleus, where it binds to antioxidant response elements (AREs), thus activating the transcription of its target genes [88]. P62/SQSTM1, a multidomain protein that regulates autophagy, has been linked to inflammation, apoptosis, and age-related pathologies. In RPE cells, p62 promotes autophagy and simultaneously enhances a Nrf2-mediated antioxidant response to protect against acute oxidative stress and mediate anti-inflammatory effects via the inhibition of the NK-*κ*B pathway. It appears that the role Nrf2 plays in autophagy, especially through interactions with p62, is strongly dependent on the cellular context as there are many reports suggesting that this protein acts differently depending on the cellular state [8]. Aging can lead to the downregulation of Nrf2 [1]. The administration of a p62/SQSTM1-encoding plasmid in OXYS mice decreased the incidence and severity of retinopathy and downregulated proinflammatory cytokines [36]. All of this data suggests that autophagy, cellular senescence, and inflammation can be linked through p62/SQSTM1 and that p62/SQSTM1 can be used as a target for the improvement of autophagy, the inhibition of retinal inflammation, and the antiaging of RPE cells.

SIRT6 and autophagic markers are upregulated in the RPE cells of aged mice. Intravitreal injections of A*β* activated SIRT6, autophagy, and inflammation. Silencing SIRT6 led to the decreased expression levels of Beclin1, ATG5, and LC3. Using 3-MA to inhibit autophagy mediated by A*β* led to decreased levels of IL-1*β*, IL-6, IL-8, IL-12b, NLRP3, and TNF-*α* [77], indicating that autophagy dysfunction resulted in the inhibition of inflammation. A2E, a major component of toxic lipofuscin that has been implicated in AMD, is deposited in RPE cells with age and can secrete inflammation-associated and angiogenic factors. The continuous incubation of RPE cells with A2E induced autophagy through the AKT/mTOR pathway and decreased cell viability in a concentration- and time-dependent manner. The application of 3-MA decreased the number of autophagosomes and LC3 puncta induced by A2E, increased the inflammation-associated expression levels of proteins, including ICAM, IL-1*β*, IL-2, IL-6, IL-8, IL-17A, IL-22, and SDF-1, and upregulated VEGFA expression. In contrast, rapamycin augmented A2E-mediated autophagy and attenuated the protein expression of inflammation-associated and angiogenic factors [21], indicating that autophagy dysfunction was accompanied by the upregulation of inflammatory responses. In addition, intracellular protein accumulation and autophagy inhibition can mediate NLRP3 activation and Alu RNA accumulation in RPE cells [89–91], thus activating inflammation [8, 18]. Therefore, changes to the autophagy and inflammatory responses are not unidirectional, and autophagy dysfunction can be accompanied by either the promotion or inhibition of inflammation.

With the depletion of glutathione (GSH) from RPE cells, increased autophagy and SIPS activation were apparent, as reflected by increased LC3 expression levels, autophagic vacuoles, and autophagic flux and an increased percentage of SA-*β*-positive cells, SAHFs, and cell cycle arrest at the G_1_ phase, indicating that SIPS and increased autophagy occurred simultaneously. However, the inhibition of autophagy with 3-MA promoted SIPS whereas inducing autophagy with rapamycin attenuated SIPS [92].

In summary, autophagy dysfunction, cellular senescence, and abnormal immune-inflammatory responses interact with each other and jointly participate in and promote AMD.

## 6. Conclusion

AMD is a blinding disease caused by genetic and environmental factors. The roles of autophagy dysfunction in RPE cells, cellular senescence, and abnormal immune-inflammatory responses have been recognized in AMD. The relationships among these three processes can be described as both stimulating and restrictive. Autophagy dysfunction in RPE cells leads to clearance system abnormalities. Cellular senescence leads to cell dysfunction and the promotion of senescence among neighboring cells. Abnormal immune-inflammatory responses lead to chronic retinal inflammation. Autophagy dysfunction can accelerate the senescence of RPE cells, while either promoting or inhibiting inflammation. In conclusion, if improved autophagy, alleviated cellular senescence, and the inhibition of abnormal retinal immune-inflammation responses can be achieved simultaneously, it may be possible to delay the progress of AMD and to obtain better clinical efficacy. At present, these three antiaging strategies have achieved good results when applied to atherosclerosis, pulmonary fibrosis, and osteoarthritis. Although there is currently no relevant application of these strategies for AMD, the use of antiaging strategies for AMD prevention and treatment is expected to achieve a new breakthrough in the future.

## Figures and Tables

**Table 1:** Candidate senotherapies.

Cell/agent	Target (or targets)	Target SNC types	Refs
*Immune-mediated SNC clearance*			
NK cells	NKG2D	IMR-90 cells, cancer cells	[48, 50]
Macrophages	oxPCCD36	SNCs in atherosclerotic plaques	[51]
Monocytes	MIF-CXCR2 axis	SNCs in atherosclerotic plaques	[49]
	CD44	HUVECs	[28]
Ipilimumab	CTLA-4, PD1	Cancer cells	[52]
*Senolysis*			
D	p21^CIP1/WAF1^, tyrosine kinases	Fat precursor cells	[54]
KKKQ	PI3K	Human endothelial cells, mouse BMSCs	[54]
D+Q	p21^CIP1/WAF1^, p16^INK4A^ BCL-Xl, PAI-2, SASP	MEFs, IMR-90 cells	[54–56]
ABT-263	BCL-2, BCL-W, BCL-Xl	MEFs, IMR-90 cells, HUVECs	[27, 28, 53]
ABT-737	BCL-W, BCL-Xl	MEFs, IMR-90 cells, HUVECs	[27, 28, 53]
FOXO4-related-peptide	p53	IMR-90 cells	[61]
AP20187	p16^INK4A^	Mouse BMSCs	[62]
*SASP neutralization*			
Rapamycin	mTOR1	IMR-90 cells, MEFs	[28, 29, 53]
Metformin	I*κ*B, IKK*α*/*β*	IMR-90 cells	[63]
JAK1/2 inhibitors	JAK1/2	Human primary preadipocytes, HUVECs	[65]
UBX0101	SASP factors	Chondrocytes	[27, 66]
